# Cleidocranial dysplasia syndrome with epilepsy: a case report

**DOI:** 10.1186/s12887-019-1472-0

**Published:** 2019-04-08

**Authors:** Yimei Ma, Fumin Zhao, Dan Yu

**Affiliations:** 1 Department of Pediatrics, West China Second University Hospital, Sichuan University, Chengdu, Sichuan 610041 People’s Republic of China; 2 Department of Radiology, West China Second University Hospital, Sichuan University, Chengdu, 610041 Sichuan China; 3 Key Laboratory of Birth Defects and Related Diseases of Women and Children (Sichuan University), Ministry of Education, Chengdu, 610041 Sichuan China

**Keywords:** Cleidocranial dysplasia, RUNX2

## Abstract

**Background:**

Cleidocranial dysplasia is a rare autosomal dominant disorder resulting in skeletal and dental abnormalities due to the disturbance in ossification of the bones. The prevalence of CCD is one in a million of live births, and epileptic seizures are rarer in this disease.

**Case presentation:**

Herein, we present a case of a 10-year-old girl, who not only suffered with cleidocranial dysplasia, but experienced frequent seizures. We initiated an anti-epileptic treatment for this patient with dose adjustments to her weight of levetiracetam (10 mg/kg, bid) for 3 months. The epileptic seizures were controlled, but the intelligence level and control of epilepsy need to be followed up for a longer duration.

**Conclusions:**

In clinical practice, if a patient has unusual facies, typical clavicle defect, skull bone enlargement, and unclosed anterior fontanelle, we should consider the possibility of cleidocranial dysplasia, genetic detection are helpful to make a confirmed diagnosis. In such cases, early diagnosis and treatment is important to correct deformities and improve the quality of life of patients.

## Background

Cleidocranial dysplasia is a rare autosomal dominant hereditary skeletal disease (MIM number is 600211). A few of these cases were familial and most were sporadic. There was no significant difference in the incidence between males and females, and the clinical incidence was 1:1000000 [1]. The main manifestations of CCD are systemic skeletal and teeth dysplasia. The typical symptoms include: incomplete or absent development of one or both clavicles, the formation of pseudo joints, shoulders drooping, shoulder joint hypermobility; delayed closure or unclosed anterior fontanelle, widening of cranial sutures, cranial ectasia; retention of deciduous teeth, delayed sprouting of permanent teeth, malformation of roots and tooth cysts with supernumerary teeth [2]. Systemic manifestations include: pigeon chest or conical shape of chest, smaller scapula, widened pubic symphysis gap, Short stature, repeated infection of respiratory tract and ear canal [3, 4]. The incidence of the disease is very low, and combined with seizures are rarer. In this paper, the clinical data of a patient with CCD accompanied by epileptic seizure were reported. Laboratory, imaging studies and related literature were reviewed to discuss the clinical manifestations and genetic characteristics of the disease to improve the clinician’s understanding and knowledge in regard to this specific congenital disorder.

## Case presentation

**Fig. 1:**
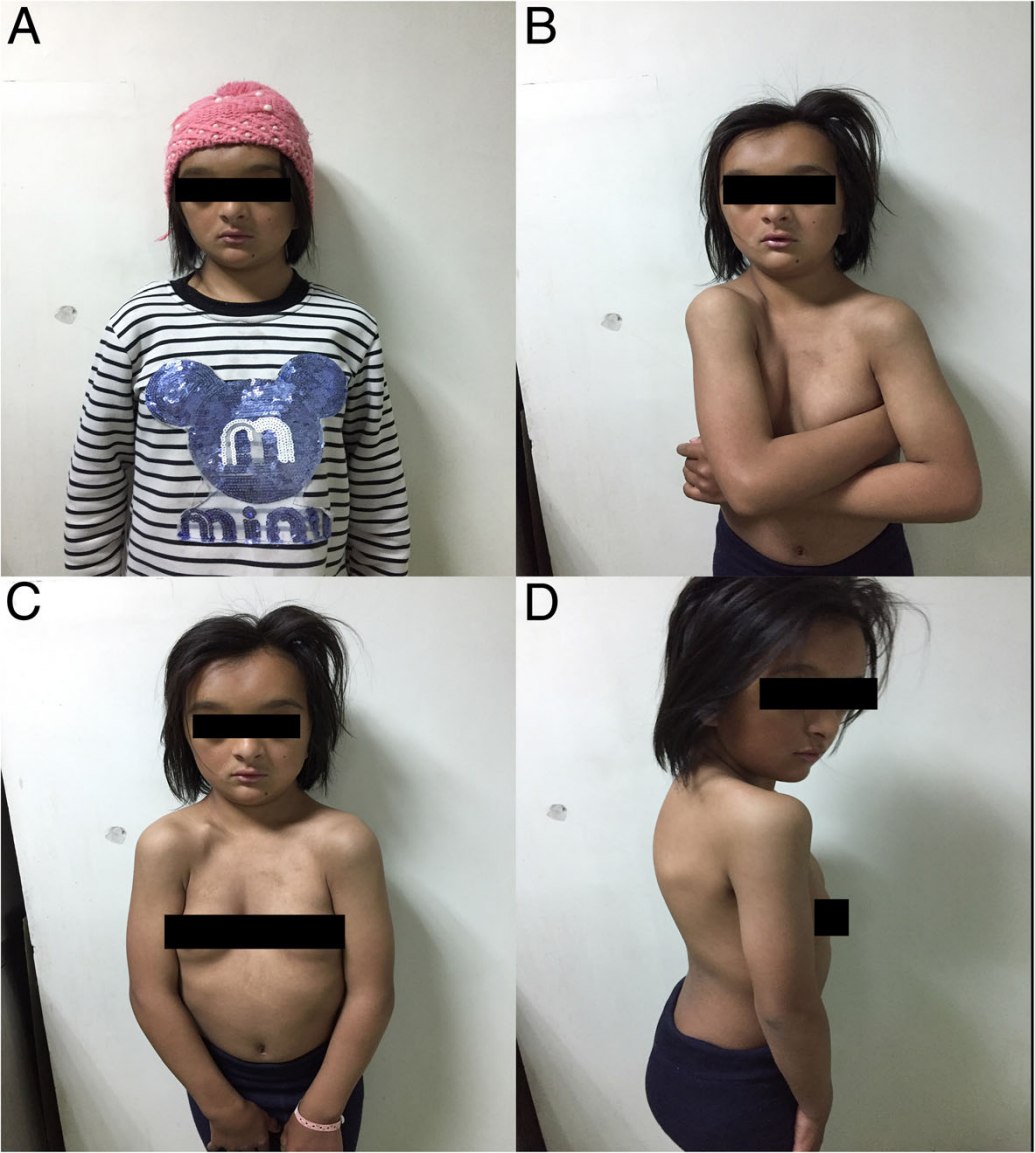
Physical examination showed obvious abnormal development in patient. (**a**: unusual facies; **b**: right shoulder towards the midline; **c** & **d**: funnel chest)

**Fig. 2:**
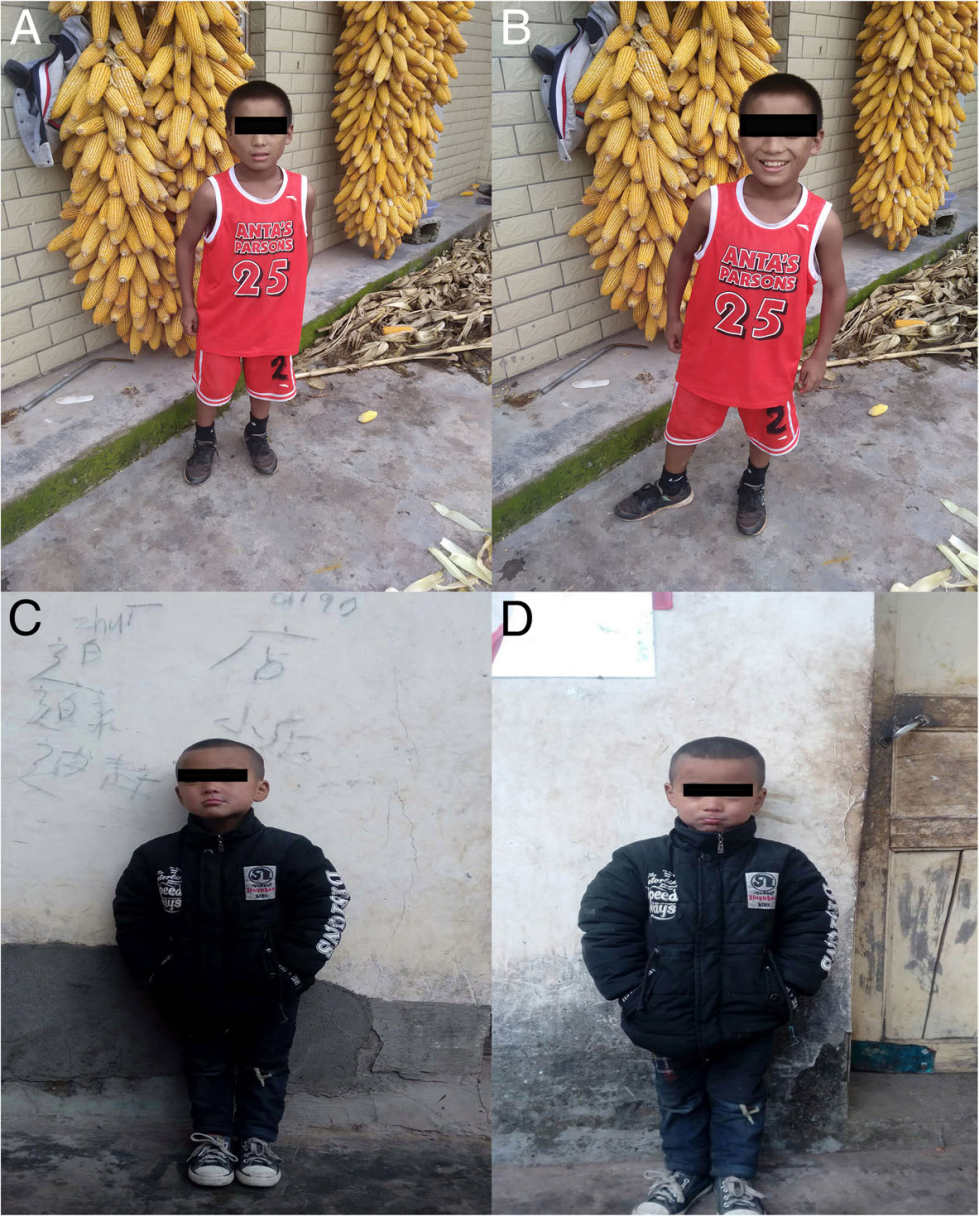
No obvious abnormalities in the development of siblings. Brother. (**a** & **b**: elder brother; **c** & **d**: younger brother)

**Fig. 3:**
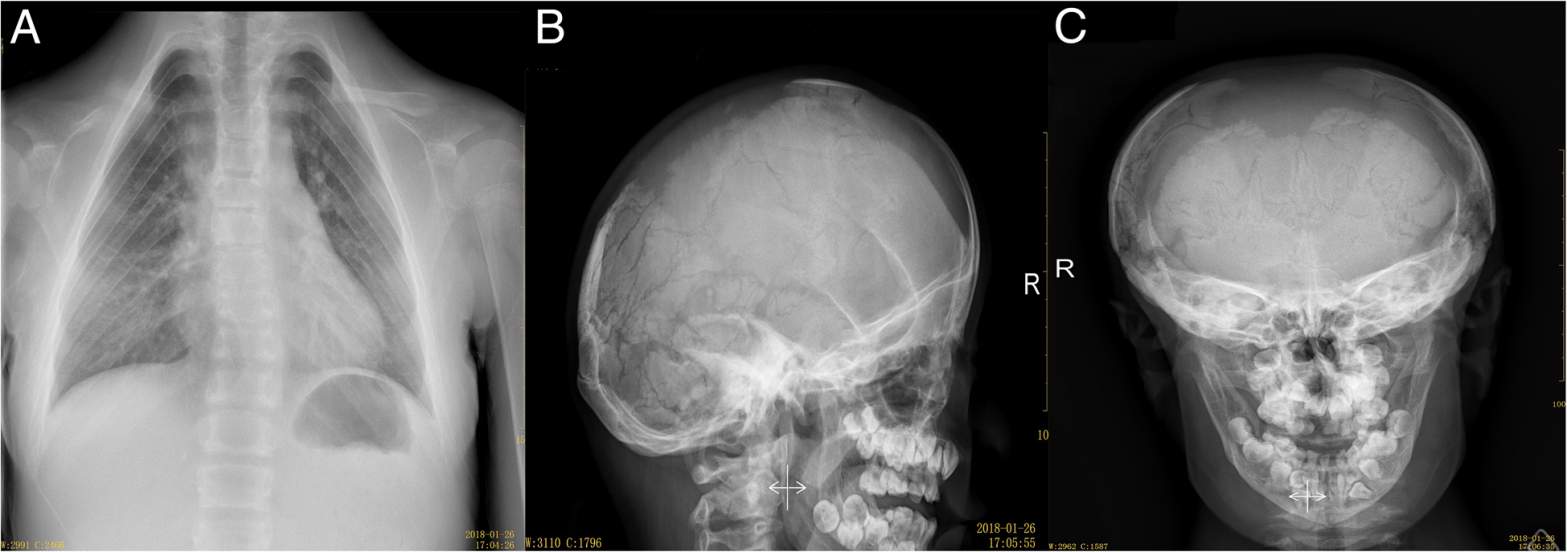
Plain radiographs showing multiple skeletal abnormalities. **a**: chest radiograph showing bilateral asymmetry clavicles with partial absence of the right; **b** & **c**: anteroposterior and lateral cephalograms showing the broad sagittal and coronal sutures, delayed closure of the anterior fontanel, numerous wormian bones, hypoplastic maxilla and unerupted permanent teeth

**Fig. 4:**
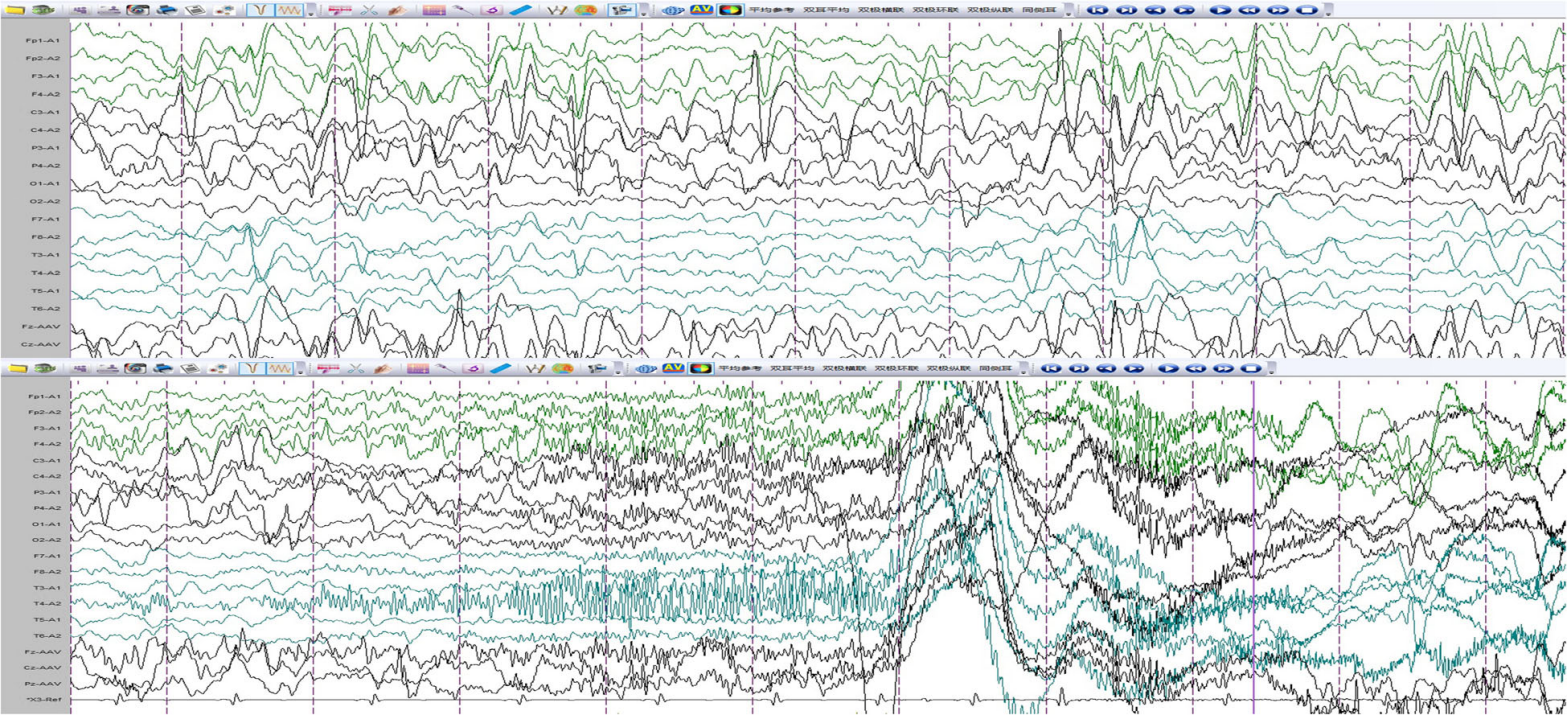
Electroencephalogram: abnormal school-age electroencephalogram: multi-focal cusp, sharp and slow wave were frequently issued., The right central, top, and mid-temporal regions show pointed and spiked rhythms repeatedly released, which is considered as complex partial seizures

**Fig. 5:**
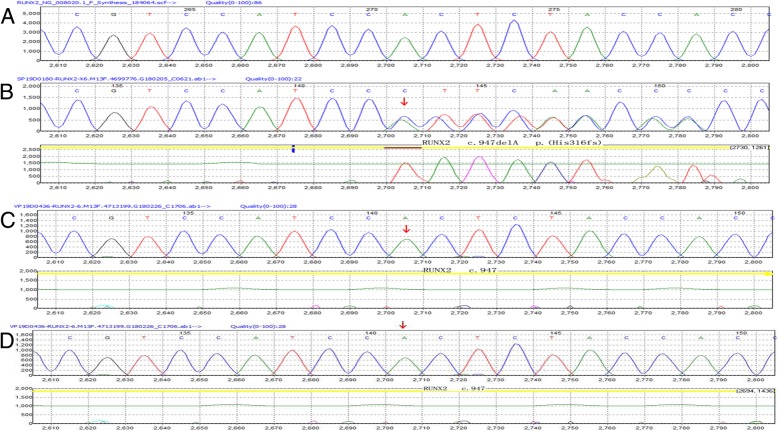
Sequencing of the RUNX2 gene: c.947delA p. (His316fs) . This disease is an autosomal dominant inheritance. The child has a heterozygous mutation whose parents are normal. This mutation is a frameshift mutation (the RUNX2 translation protein has a disorder in the coding of the amino acid residue His at position 316). This mutation is expected to cause the encoded protein to be truncated and lose its normal function. (**a**: reference; **b**: patient; **c**: the father; **d**:the mother)

A 10 years and 6 months old girl, was admitted to hospital due to “epileptic seizures for five months”, which manifested as an involuntary nodding movement accompanied by loss of consciousness, with no fever, limb stiffness, cyanosis, salivation and incontinence. During the early period of illness, these symptoms lasted for about 10 s - with a frequency of about 2 episodes per day. Then it gradually increased to 30 s to 1 min before spontaneous cessation, with a frequency of about 4–5 times a day. The patient was treated with “carbamazepine and vitamin B6” but there was no obvious improvement in symptoms or progression of illness. Physical examination done at time of admission: T 36.8 °C, P 89 beats /min, R 19 beats / min, BP 109/68 mmHg. Weight 27 kg, height 125 cm. The patient is positive for special type of facieswitha flat nose, wide-set eyes, micrognathia, deciduous and misaligned teeth, and 9 maxillary and mandibular teeth. Head circumference is 52 cm.The anterior fontanelle is open, approximately 4 × 4 cm size, and soft on palpation. The sagittal and coronal sutures are unclosed. The width of the sagittal suture is about 6 cm. The width of the coronal suture is about 0.5 cm. They are all soft and flat, without tenderness. No résistance was felt in the neck. Defects can be observed in the right clavicle, bilateral shoulders can reach the midline. The thoracic cavity hollows and changes like a funnel. A brown patch which is approximately 2.5 × 1.0 cm in size is visible on the left wrist and a light brown patch of approximately 3.5 × 4.0 cm is visible on the right wrist. The double knee valgus is deformed in an “X” shape with no limitation of activity. There were no abnormalities in the spine and joints, and the muscle strength and muscle tones of the limbs were normal. The pathological reflex were negative (Fig. 1). The child raised his head at 3 months old, crawled and rolled over at 7 months old, walked alone and called daddy and Mommy at 1 year old. At present, she is in the third grade of elementary school, she can answer simple questions but cannot add or subtract within 10. The funnel chest was found a week after birth, and the anterior fontanelle was not closed at 2 years old, none of them were treated. The child has two brothers, both healthy, with no obvious abnormalities in development (Fig. 2). laboratory examination: blood routine: WBC 4.6 × 10^9^/L, N 34.9%, HB 134 g/L, PLT 281 × 10^12^/L; Blood ammonia: 40 umol/L; Vitamin D determination: 12.8 ng/ml; Kidney and kidney function, electrolytes, blood gas analysis, glucose, pyruvate, β - hydroxybutyrate, calcium/phosphate/alkaline phosphatase, urination and defecation were both normal. Electrocardiogram is normal overall. X-ray examination showed: Asymmetry in Bilateral clavicles, a third of the right lateral clavicle is absent. The cranium is higher, the cranial suture are widened, and the anterior fontanelle was not closed. The bone structure of the cranial plate is not completely developed. There are many suture bones in the skull. (Fig. 3). Video EEG: Abnormal: multi-focal sharp wave, sharp and slow wave were frequent issued (Fig. 4). Skull clavicle dysplasia syndrome RUNX2 gene sequencing suggested RUNX2 c.947delA p. (His316fs) heterozygous, pathogenic frameshift mutations (Fig. 5).

## Discussion and conclusions

CCD is also known as Marie and Sainton ‘s disease, Schlotzhauer-Marie-Sainton syndrome, osteogenesis imperfecta, which was first described by Pierre Marie and Paul Sainton in 1898 [5]. RUNX2 gene is a CCD gene that is located on chromosome 6p21 and is a 130 kb length protein. It encodes the transcription factors required for chondrocyte proteins and dentin, influences the differentiation and maturation of osteoblasts, and affects intramembranous and endochondral ossification [4, 6–8]. In our case, the sequencing of the RUNX2 gene indicate c.947delA p. (His316fs) mutation in patient while her parents’ are normal. This mutation is a frameshift mutation (the RUNX2 translation protein has a disorder in the coding of the amino acid residue His at position 316). This mutation is expected to cause the encoded protein to be truncated and lose its normal function. In addition to the typical dystrophy of the cranial clavicle and various vertebral and dental abnormalities, recurrent ear infections and hearing loss are common in CCD due to maxillofacial hypoplasia and osteogenic deficiencies [3]; most patients have chest malformations, some severe cases can lead to an early respiratory distress in infants [4]; some case also indicate that CCD patient are more likely to suffer from hematological disease [9–11]. Osteopenia is present in most patients due to osteogenesis and ossification [5]; CCD patients are often of short stature, their birth length is usually normal, but their height is below the two percentiles between 4 and 8 years old. The final average height of the male is 165 cm (±8 cm) and the female is 156 cm (±10 cm) [12]. The motor development may be of slightly delayed onset (age of walking), but in most cases, mental development is normal [13].

This patient had a typical clavicle defect, skull bone enlargement, and unclosed anterior fontanelle, with funnel chest which conforms to the typical CCD performance and the results of genetic sequencing, confirmed this diagnosis. Several neurological disorders associated with CCD have been reported such as mental retardation, spastic paresis etc. [14]. Seizures associated with this disease are very rare. The patient was diagnosed with epilepsy at 5 years of age but was not on regular treatment. Most of the CCD patients had normal intelligence, but the mental development of this patient was significantly backward compared with children of the same age. As a student of the third grade of elementary school, she cannot read and cannot perform simple addition and subtraction in less than 10. Her cranial magnetic resonance imaging (MRI) showed bilateral parietooccipital atrophy and periventricular leukomalacia. According to her medical history, the mental development delay may be related to the brain damage and poor control of epilepsy. Analysis of the Literature on CCD patients with epilepsy, we found that there were few related literatures. Most of the articles were case reports which had been published over 10 years ago. B. M. Tress’s research found that among the patients with epilepsy, there were 3 cases in which this was secondary to an abscess, 2 with cerebral angiomas and 2 with telangiectasia [15]. Oyer, CE reported a 15-day-old female neonate with CCD. Her postnatal course was characterized by seizures and recognition of hydrocephalus during the first day of life [16].

This patient had no family history of epilepsy, but cranial MRI suggested sequela of brain injury. So the etiology of epilepsy maybe brain damage. Consistent with the literature, the occurrence of epilepsy may be related to brain structural abnormalities and damage. But the number of cases are limited, further research is necessary in the future. After the patient was admitted to the hospital, oral levetiracetam (10 mg/kg, bid) was used to control seizure and she received supportive treatment [17, 18]. The intelligence level and control of epilepsy need to be followed up for a longer period of time [19–21].

In clinical practice, if one patient has unusual facies, typical clavicle defect, skull bone enlargement, and unclosed anterior fontanelle, we should consider the possibility of cleidocranial dysplasia, in such cases, early diagnosis and treatment is important to correct deformities and improve the quality of life of patients.

## Abbreviations

BP: Blood pressure
CCD: Cleidocranial dysplasia
P: Pulse
R: Respiratory
T: Temperature
